# Ultraviolet radiation-induced tumor necrosis factor alpha, which is linked to the development of cutaneous SCC, modulates differential epidermal microRNAs expression

**DOI:** 10.18632/oncotarget.7595

**Published:** 2016-02-22

**Authors:** Ashok Singh, Estelle Willems, Anupama Singh, Bilal Bin Hafeez, Irene M. Ong, Suresh L. Mehta, Ajit Kumar Verma

**Affiliations:** ^1^ Department of Human Oncology, Wisconsin Institutes for Medical Research, Paul P. Carbone Comprehensive Cancer Center, School of Medicine and Public Health, University of Wisconsin, Madison, WI, USA; ^2^ Biostatistics and Medical Informatics, Medical Science Center, University of Wisconsin, Madison, WI, USA; ^3^ Department of Neurological Surgery, University of Wisconsin, Madison, WI, USA

**Keywords:** microRNA, TNFα, SCC, UVR

## Abstract

Chronic exposure to ultraviolet radiation (UVR) is linked to the development of cutaneous squamous cell carcinoma (SCC), a non-melanoma form of skin cancer that can metastasize. Tumor necrosis factor-alpha (TNFα), a pro-inflammatory cytokine, is linked to UVR-induced development of SCC. To find clues about the mechanisms by which TNFα may promote UVR-induced development of SCC, we investigated changes in the expression profiling of microRNAs (miRNA), a novel class of short noncoding RNAs, which affects translation and stability of mRNAs. In this experiment, TNFα knockout (TNFα KO) mice and their wild type (WT) littermates were exposed to acute UVR (2.0 kJ/m^2^) and the expression profiling of epidermal miRNA was determined 4hr post UVR exposure. TNFα deletion in untreated WT mice resulted in differential expression (log fold change>1) of seventeen miRNA. UVR exposure in WT mice induced differential expression of 22 miRNA. However, UVR exposure in TNFα KO mice altered only two miRNAs. Four miRNA, were differentially expressed between WT+UVR and TNFα KO+UVR groups. Differentially expressed selected miRNAs were further validated using real time PCR. Few of the differentially expressed miRNAs (miR-31-5p, miR-196a-5p, miR-127-3p, miR-206-3p, miR-411-5p, miR-709, and miR-322-5p) were also observed in UVR-induced SCC. Finally, bio-informatics analysis using DIANA, MIRANDA, Target Scan, and miRDB algorithms revealed a link with major UVR-induced pathways (MAPK, PI3K-Akt, transcriptional mis-regulation, Wnt, and TGF-beta).

## INTRODUCTION

Ultraviolet radiation (UVR) is a potent environmental carcinogenic agent, and its chronic exposure leads to cutaneous Squamous Cell Carcinoma (cSCC) [1]. Cutaneous SCC is the second most common non melanoma skin cancer (NMSC) with approximately 250,000 new cases per year [2]. Cutaneous SCC originates from the epidermal keratinocytes [2]. UVR exposure can directly damage the cellular DNA [3, 4]. UVR induces DNA lesions, which remains unrepaired and accumulated on replication resulting in the expansion of initiated clones. The whole process is facilitated by aberrant gene expression during initiation process of carcinogenesis. Recently, it has been observed that >60% of human protein coding genes are regulated by microRNAs (miRNAs), a novel class of regulators [5].

MiRNAs are small non-protein coding RNAs and endogenous in their origin. The nucleotide size of these miRNAs ranges from 19-22. These small endogenous regulators play important roles in post-transcriptional process of various protein coding genes [6, 7]. Such miRNA regulation is mediated by the binding of miRNA with a partially complimentary target site within the 3′ untranslated region (3′-UTR) of their target mRNA [5]. Interestingly, a single miRNA is able to suppress several target mRNAs; and in turn be targeted by multiple miRNAs [8]. Various components of miRNA pathways are found to be affected in epithelial skin cancer [9-12]. Also, the UVA and UVB irradiation differentially regulate miRNA expression in human primary keratinocytes [13]. Identification of novel miRNAs involved in UVR-induced skin cancer [14] may be useful as early diagnostic markers, and to precisely define cancer stages and its progression. However, the literature for UVR-induced miRNAs in cutaneous skin cancer is limited [15].

UVR exposure in mice skin results in elevated release of various pro-inflammatory cytokines including tumor Necrosis Factor alpha (TNFα) [16, 17]. It has been observed that cutaneous damage due to UVR is less in TNFα knock out (TNFα KO) mice compared to their wild type [17, 18]. Additionally, TNFα KO and TNFα receptor KO mice are resistant to development of skin cancer elicited by repeated UVR exposure [19, 20]. However, a precise molecular mechanism by which TNFα signals UVR-induced skin carcinogenesis is not understood clearly.

In this communication, we determined whether TNFα deletion in mice affects UVR-induced expression profile of epidermal miRNAs. We report for the first time: a) UVR-induced expression profile of epidermal miRNAs in wild type (WT) and TNFα KO mice, b) validation of the differentially expressed epidermal miRNAs using real time PCR, c) expression pattern of miRNAs in UVR-induced SCC samples, and d) bio-informatics analysis of UVR-induced epidermal miRNAs and their targeted genes.

## RESULTS

### Differential expression of miRNAs responsive to acute UVR exposure in the epidermal skin of WT and TNFα KO mice

To find clue about the UVR-induced miRNA modulation, the mice were divided into two groups. The first group was untreated (WT, TNFα KO), and the second group (WT+UVR, TNFα KO+UVR) was exposed to acute UVR (single dose, 2.0 kJ/m^2^). Total RNA from the whole skin was isolated for global miRNA profiling 4hr post UVR. We found differential expression (log fold change>1) of 22 miRNAs between the WT and WT+UVR group and 17 miRNAs between the WT and TNFα KO group (Tables 1, 2). Two miRNAs were differentially expressed between TNFα KO and TNFα KO+UVR, and four miRNAs between WT+UVR and TNFα KO+UVR groups (Tables 3, 4). A comparison between WT and WT+UVR group revealed the up regulation of six miRNAs (miR-31-5p, miR-31-3p, miR-709, miR-5617, miR-691, and miR-185-3p) and down regulation of sixteen miRNAs (see Table 1). There were two highly suppressed miRNAs (log fold change =2) miR-196a-5p and miR-196b-5p following acute UVR exposure compared to untreated WT mice. Also, miRNAs miR-31-5p (log FC =2) and miR-31-3p (log FC =1.7) were up-regulated due to acute UVR treatment in WT mice compared to untreated littermates. Moreover, a comparison between WT and TNFα KO mice, revealed the up regulation of one microRNA (miR-3065-3p) and down regulation of sixteen miRNAs (Table 2). Two miRNAs miR-196b-5p and miR-206-3p were up regulated between TNFα KO and TNFα KO+UVR.

**Table 1:** Differential expression pattern of UVR-induced miRNAs in WT and their UVR treated littermates

microRNAs	Location on chromosome	WT	WT+UVR	Log Fold Change	Adjusted *p*-value	Total # of gene targets
miR-127-3p	chr12: 109592846-109592915 [+]	1.089	−0.401	−1.49	1.11E-06	12
miR-136-3p	chr12: 109595327-109595388 [+]	1.027	−0.48	−1.507	2.04E-06	32
miR-136-5p	chr12: 109595327-109595388 [+]	1.246	−0.431	−1.677	1.11E-06	275
miR-154-5p	chr12: 109738433-109738498 [+]	0.811	−0.283	−1.094	2.04E-06	201
**miR-185-3p**	chr16: 18327401-18327465 [−]	−0.853	0.225	1.078	3.10E-05	203
miR-196a-5p	chr11: 96265164-96265265 [+]	1.441	−1.405	−2.846	1.11E-06	137
miR-196b-5p	chr6: 52230081-52230165 [−]	1.223	−0.962	−2.185	1.11E-06	134
miR-206-3p	chr1: 20679010-20679082 [+]	0.602	−0.492	−1.094	2.41E-06	336
**miR-31-3p**	chr4: 88910557-88910662 [−]	−0.823	0.897	1.72	1.11E-06	100
**miR-31-5p**	chr4: 88910557-88910662 [−]	−1.07	0.962	2.031	1.11E-06	263
miR-335-5p	chr6: 30741299-30741396 [+]	1.152	−0.175	−1.327	1.80E-05	237
miR-376a-3p	chr12: 109723781-109723848 [+]	1.016	−0.382	−1.398	2.90E-05	24
miR-376b-5p	chr12: 109723458-109723539 [+]	0.945	−0.146	−1.091	2.04E-06	225
miR-377-3p	chr12: 109740510-109740577 [+]	0.919	−0.338	−1.256	2.04E-06	517
miR-379-5p	chr12: 109709060-109709125 [+]	1.172	−0.292	−1.464	1.11E-06	73
miR-411-5p	chr12: 109710175-109710256 [+]	1.091	−0.442	−1.533	1.43E-06	157
miR-434-3p	chr12: 109594506-109594599 [+]	0.954	−0.344	−1.298	5.37E-06	155
miR-434-5p	chr12: 109594506-109594599 [+]	1.08	−0.394	−1.475	2.04E-06	24
miR-511-3p	chr2: 14261003-14261081 [+]	0.795	−0.444	−1.239	2.04E-06	467
**miR-5617-5p**	chrX: 20863126-20863182 [−]	−1.015	0.138	1.152	3.75E-06	267
**miR-691**	chr16: 74341990-74342067 [−]	−0.897	0.242	1.139	2.06E-06	413
**miR-709**	chr8: 84086099-84086186 [+]	−1.091	0.249	1.34	2.32E-06	445

The Tables (1-4) are showing the only miRNAs where the absolute value of the log fold change is >1 (log fold change of 1 equals a fold change of 2(2^1=2). For each comparison the fold change expression is listed as log2 transformed values in the logFC column. Bold texts in microRNA tables are showing the up regulated miRNAs. Last column is showing the total number of target genes by the miRNA using miRDB database. Abbreviations: FC = fold change, mmu = denotes name code for miRNA in mouse species.

**Table 2:** Differential expression pattern of miRNAs in WT and TNFα KO mice

microRNAs	Location on chromosome	WT	TNFα KO	Log FC	Gene targets
miR-127-3p	chr12: 109592846-109592915 [+]	1.089	−0.622	−1.711	12
miR-136-3p	chr12: 109595327-109595388 [+]	1.027	−0.488	−1.515	32
miR-136-5p	chr12: 109595327-109595388 [+]	1.246	−0.631	−1.877	275
miR-154-5p	chr12: 109738433-109738498 [+]	0.811	−0.442	−1.253	201
miR-196a-5p	chr11: 96265164-96265265 [+]	1.441	−1.284	−2.724	137
miR-196b-5p	chr6: 52230081-52230165 [−]	1.223	−0.757	−1.979	134
miR-335-5p	chr6: 30741299-30741396 [+]	1.152	−0.132	−1.284	237
miR-376a-3p	chr12: 109723781-109723848 [+]	1.016	−0.807	−1.823	24
miR-376b-5p	chr12: 109723458-109723539 [+]	0.945	−0.222	−1.167	225
miR-377-3p	chr12: 109740510-109740577 [+]	0.919	−0.449	−1.368	517
miR-379-5p	chr12: 109709060-109709125 [+]	1.172	−0.616	−1.788	73
miR-411-5p	chr12: 109710175-109710256 [+]	1.091	−0.609	−1.7	157
miR-434-3p	chr12: 109594506-109594599 [+]	0.954	−0.439	−1.393	155
miR-434-5p	chr12: 109594506-109594599 [+]	1.08	−0.51	−1.59	24
**miR-3065-3p**	chr11: 120014767-120014853 [+]	−0.699	0.745	1.444	253
miR-541-5p	chr12: 109742409-109742498 [+]	0.732	−0.574	−1.306	164
miR-322-5p	chrX: 53054255-53054349 [−]	0.66	−0.368	−1.028	613

**Table 3:** Differential expression pattern of UVR-induced miRNAs in TNFα KO mice

microRNAs	Location on chromosome	TNFα KO	TNFα KO + UVR	Log FC	Gene targets
miR-196b-5p	chr6: 52230081-52230165 [−]	−1.284	0.714	1.998	134
miR-206-3p	chr1: 20679010-20679082 [+]	−0.757	0.522	1.278	336

**Table 4:** Differential expression pattern of UVR-induced miRNAs in WT and TNFα KO mice

microRNAs	Location on chromosome	TNFα KO+UVR	WT+UVR	log FC	Gene targets
miR-196b-5p	chr6: 52230081-52230165 [−]	0.714	−1.405	2.12	134
miR-206-3p	chr1: 20679010-20679082 [+]	0.522	−0.962	1.484	336
miR-31-3p	chr4: 88910557-88910662 [−]	−0.831	0.897	−1.729	100
miR-31-5p	chr4: 88910557-88910662 [−]	−0.992	0.962	−1.954	263

**Figure 1:**
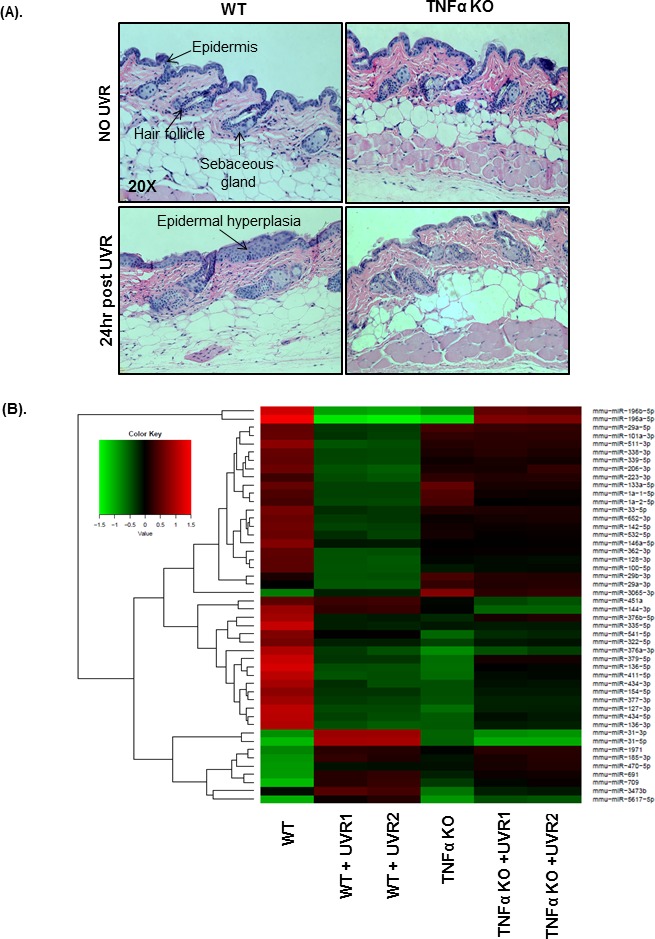
Skin histology and hierarchical clustering of miRNA expression **A.** is showing the representative pictures of skin histology and UVR-induced hyperplastic epidermis in the skin of WT, and TNFαKO mice. Briefly, mice were exposed once to UVR (2.0 kJ/m^2^), and were sacrificed at 24 h post UVR. For histochemistry, mice skin specimen (*n* = 3 each) were processed for H & E staining. All H & E pictures were taken in a Nuance bright field microscope at 20X magnification. The **B.** is showing the clustering performed on the top forty eight miRNAs with highest standard deviation. The values of normalized log ratio were used for the analysis. The heat map diagram is showing the result of a two-way hierarchical clustering of miRNAs in mice samples (*n* = 2 each). The complete-linkage method and the Euclidean distance measure are used for the miRNA clustering. Each row in the clustering diagram represents miRNA and each column represents a mice sample. The left side of the **B.** is showing clustering tree. The color key in top left of the **B.** is showing the relative expression level of miRNAs. Red and green colors in the clustering diagram are showing higher and lower expression of miRNAs than the reference channel respectively.

Interestingly, miR-3065-3p, miR-541-5p, and miR-322-5p are exclusively and differentially expressed between WT and TNFα KO mice skin following acute UVR exposure (Table 2). Also, miR-185-3p is not differentially expressed between WT and TNFα KO mice. A comparison between WT vs WT+UVR, and TNFα vs TNFα KO mice revealed that there are common miRNAs, which are differentially expressed among these groups. Literature mining revealed that the differentially expressed microRNAs miR-196a-5p, miR-127-3p and miR-206-3p are found to be expressed in hair follicles of mice skin [21]. Also, we observed increased epidermal hyperplasia in 24hr post UVR treated WT mice compared to TNFα KO (Figure 1A). There was no hyperplasia in TNFα KO mice. Hierarchical clustering analysis based on the 48 differentially regulated miRNAs completely distinguished WT and TNFα KO mice from their UVR treated littermates (Figure 1B).

**Figure 2:**
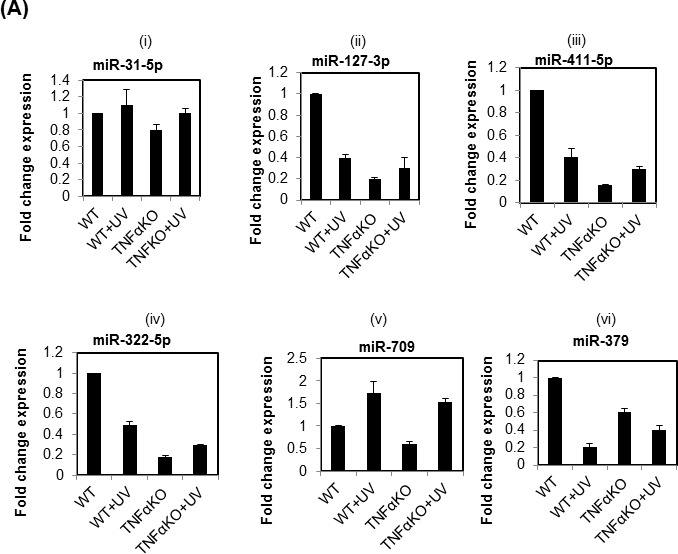

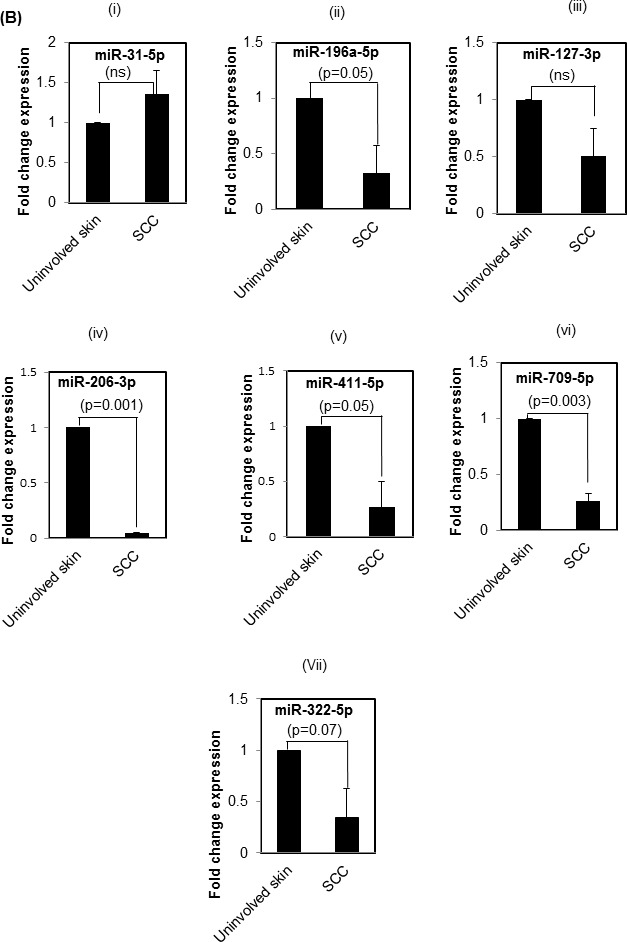
Validation of differentially expressed miRNAs using real time PCR in WT, TNFα KO and their respective UVR treated mice group **A.** is showing the real time expression pattern of miR-31-5p, miR-127-3p, miR-411-5p, miR-322-5p, miR-709, and miR-379 in WT, WT + UVR, TNFα KO and TNFα KO + UVR mice. The data presented in the each bar diagram is the mean ±SE from triplicate sample in all four mice groups. The detail of the method is discussed in materials and methods section. The **B.** is showing the expression pattern of miRNAs miR-31-5p, miR-196-5p, miR-127-3p, miR-206-3p, miR-411-5p, miR-709-5p, and miR-322-5p in UVR-induced SCC samples from wild type FVB mice. Each value is the mean ±SE from three UVR-induced SCC samples (n=3). (Abbreviation: ns = non-significant).

To confirm the miRNA Array expression data, we further determined the expression pattern of selected miRNAs using SYBR green based chemistry. The expression patterns of miR-31-5p, miR-127-3p, miR-411-5p, miR-322-5p, miR-709, and miR-379-5p were similar to the microarray results in our profiling study among four mice groups namely WT, WT + UVR, TNFα KO, and TNFα KO+UVR following acute UVR treatment (Figure 2A). Only miR-3065-3p was up-regulated in TNFα KO mice compared to their WT littermates. Validation data with RT-PCR confirms the findings of global miRNA array platform.

### Differential regulation of miRNAs in SCC samples induced by UVR

In our miRNA global expression profiling study most of the miRNAs were down regulated except few of the up-regulated miRNAs (Tables 1, 2, 3). To determine the expression pattern of validated miRNAs in SCC samples harvested from FVB mice following UVR-induced carcinogenesis. Expression pattern of UVR-induced SCC was compared with the uninvolved skin harvested from the same group of mice. The miRNA miR-31-5p was up regulated in three SCC samples compared to uninvolved skin. We also determined the expression pattern of miRNAs such as miR-196a-5p, miR-127-3p, miR-411-5p, and miR-206-3p in UVR-induced SCC samples from wild type FVB mice. Some of the miRNAs were significantly down-regulated [miR-196a-5p (p=0.05), miR-709-5p (p=0.003), miR-206-3p (0.001), miR-411-5p (p=0.03)] along with others miR-127-3p, miR-322-5p in UVR-induced SCC samples (n=3) compared to the uninvolved skin (n=3) (Figure 2B).

### Bioinformatics analysis reveals dysregulation of UVR-induced target genes and their targeted pathways following miRNA alteration

To determine the functional relevance of the up- and down-regulated miRNAs following UVR, we predicted targets of these miRNAs in skin. We used four microRNA target prediction web tools namely DIANA, MIRANDA, Target Scan, and miRDB to predict the target genes modulated by UVR and TNFα (Supplementary Tables S1 and S2). Our software based prediction indicates multiple gene targets for miRNAs (Tables 1, 2, 3, 4). We found that a minimum of 12 to a maximum of 613 genes were targeted by miRNAs namely miR-127-3p and miR-322-5p respectively (Tables 1, 2, 3, 4).

**Figure 3:**
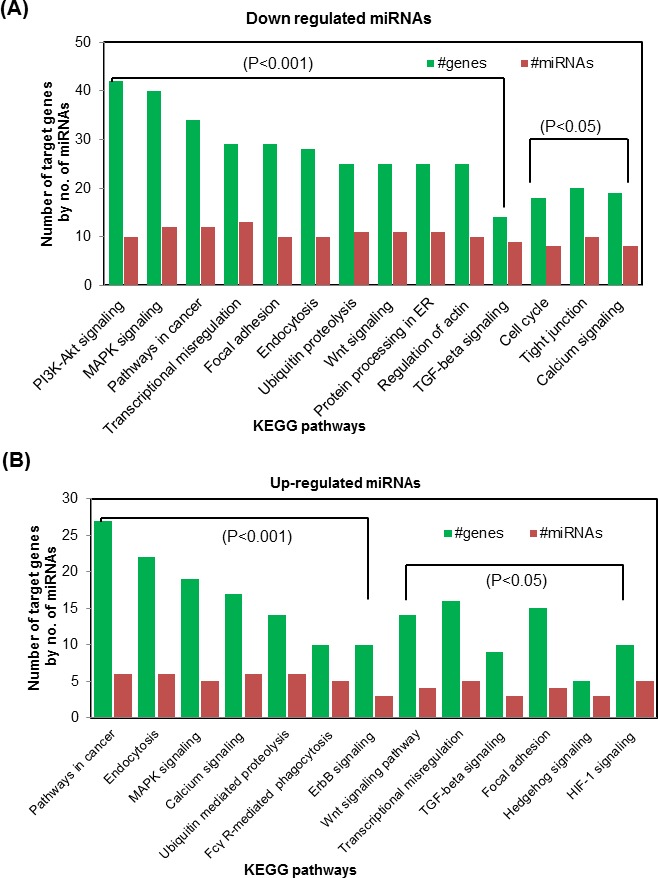
Bioinformatics analysis of differentially expressed miRNAs following acute UVR exposure in WT and TNFα KO mice **A.** and **B.** are the top KEGG pathways of biological function of the targets of down- and up-regulated mouse miRNAs that were observed to be altered at 4 hr post UVR treatment in WT and TNFα KO mice. The analysis in **A.** and **B.** is based on the combinatorial effects of down- and up-regulated miRNAs. A detail about the bio-informatics analysis is given in materials and methods section.

As it is known that miRNAs are key regulators in various biological processes and their functional outcomes, we investigated the combinatorial effect of all up- and down-regulated miRNAs in various biological pathways following acute UVR exposure. In such enrichment analysis of miRNAs, we grouped them separately to determine their functional influence in various biologically relevant pathways using DIANA miRPath v.2.0 (Figure 3A-3B). We found that the nine pathways related to transcriptional misregulation in cancer, biotin metabolism, MAPK signaling, lysine degradation, ubiquitin mediated proteolysis, cell cycle, gap junction, TGF-β signaling, and circadian rhythm, are affected due to sixteen down-regulated miRNAs between WT and WT+UVR mice; and seventeen down-regulated microRNAs between WT and TNFα KO mice. Importantly, the “transcriptional misregulation in cancer” pathway is affected by most of our differentially expressed miRNAs (miR-136-5p, miR-196a-5p, miR-196b-5p, miR-376a-3p, miR-335-5p, and miR-206-3p) significantly (p<0.001) (Figure 3A).

The combinatorial effect of six up-regulated miRNAs (miR-691, miR-709, miR-31-5p, miR-5617-5p, miR-31-3p, and miR-185-3p) leads to the following important signaling pathways such as MAPK signaling, pathways in cancer, Ca^+2^ signaling, ubiquitin proteolysis, lysine degradation, endocytosis, glycosaminoglycan biosynthesis (chondroitin sulfate and keratin sulfate), Fc gamma-mediated phagocytosis, amphetamine addiction, bladder cancer, ECM-receptor interaction, steroid biosynthesis, dopaminergic synapse, and steroid hormone biosynthesis using pathways union mode. These pathways are significantly (p=0.05 and 0.001) targeted due to acute UVR exposure between UVR-treated FVB mice and their untreated littermates. In this analysis, a maximum of nine and seven pathways are affected due to miR-185-3p, and miR-31-5p respectively. Taken together both down- and up-regulated miRNA affects some common pathways which are implicated in UVR-induced carcinogenesis.

## DISCUSSION

UVR, the component of sunlight has been linked to the development of non-melanoma skin cancer [1]. TNFα is up-regulated in response to UVR exposure. TNFα has been reported an endogenous mouse skin tumor promoter. The TNFα deficient mice are resistant to the skin cancer development induced by UVR and DMBA-TPA tumor promotion protocol [19]. But the molecular mechanism linked to the resistance to UVR is not clear. Present miRNA profiling study compares the miRNA profile among WT, TNFα KO, and their UVR-treated littermates, and further predicts the probable miRNA targets of differentially expressed miRNAs in mouse skin. We observed the differential expression of 22 miRNAs due to UVR in WT mice and 17 miRNAs between WT and TNFα KO group. Fourteen common miRNAs are differentially expressed among these four groups (Table 1-3). A total of sixteen microRNAs are down regulated in TNFα KO mice compared to their WT littermates. These miRNAs may have implication in UVR-induced resistance via suppression of oncogenic signaling. Also, the possibilities of combinatorial regulation of miRNAs in their skin micro-environment cannot be ignored in absence of TNFα. We have chosen 4hr post UVR time point for miRNA profiling as many epidermal physiological alterations takes place in epidermis at early UVR-exposure time points [22, 23]. Similarly, we can observe UVR-induced hyperplasia after 24 hr time points in mice skin [24]. It has been shown that the microRNA mediated gene regulatory network is dysregulated during the process of carcinogenesis [25]. These miRNAs contribute as an additional layer of regulators or fine tuner during gene expression. This miRNA profiling study provides novel insight for future exploration of miRNAs in connection with UVR and their functional implication in skin carcinogenesis.

In the present study, one of the miRNA miR-31-5p (>2 log FC) observed to be over expressed in microRNA profiling study and UVR-induced tumor sample in WT mice. Similarly, microRNA miR-31-3p is also over expressed (FC=1.72 log FC) in mouse skin followed by acute UVR exposure (2.0 kJ/m^2^) in initial miRNA profiling. It appears that both these mature miRNAs (miR-31-5p and -3p) originated from two opposite arm of the same pre-miRNA. The over-expression of miR-31-5p may have direct link with UVR. The increased expression of miR-31 is reported in cutaneous human SCC [10, 11]. Conversely, the miRNA-31 is found to be down regulated in primary and metastatic melanoma [26] and can pleiotropically act as an inhibitor of breast cancer metastasis [27]. Also, our bioinformatics analyses revealed that miR-31-5p target the PKCε gene, which is an endogenous photosensitizer which sensitizes the skin by enhancing UVR-induced cutaneous damage and development of cutaneous SCC [16].

A number of studies have explored the pattern of the global miRNA profiling in various cell lines and skin cancer [11, 12, 28]. In the present miRNA profiling study, most of the miRNAs are down regulated following TNFα deletion and UVR treatment. We also observed the suppression of miRNAs in SCC samples compared to uninvolved mice skin for miR-195-5p, miR-127-3p, miR-206-3p, miR-411-5p, miR-709-5p, and miR-322-5p. These observations are in corroboration with the earlier findings, where they have shown the suppression of various miRNAs in cancer [29, 30]. The repression may be due to the direct impact of acute UVR or other indirect regulations involving biogenesis pathways, epigenetic modification, and alteration in transcriptional machinery [30, 31]. We also observed the differential expression of miR-196a-5p, miR-127-3p, and miR-206-3p in our samples. These miRNAs are found to be differentially and highly expressed in skin hair follicles compared to epidermis [21]. These miRNAs may have some potential implication in the hair follicle morphogenesis and UVR signaling.

Notably, a single miRNA can target various gene targets and act on hundreds of mRNAs that contain complementary binding sites. These target genes may be the integral part of the same biological pathways. We looked at the synergistic role of up- and down-regulated miRNAs due to acute UVR to determine the pathways affected and implicated in the process of UVR-induced carcinogenesis. We found the pathways related to transcriptional misregulation in cancer, cell cycle, TGF-β signaling, MAPK signaling, Wnt signaling, Ubiquitin mediated proteolysis, PI3K-Akt signaling targeted by more than one miRNA gene in both up- and down-regulated miRNAs. Most of these signaling pathways are implicated in different types of skin cancer progression via direct as well as indirect involvement of UVR [32-36]. In our bio-informatics analysis, targeted genes and predicted pathways provide clues for further studies.

In conclusion, we determine UVR-induced expression profile of miRNAs in the skin of WT and TNFα KO mice. Bio-informatics based analysis revealed the miRNA targeted genes as well as the potential signaling pathways affected due to modulation of miRNAs following acute UVR exposure in epidermal skin. Our finding suggests that the single UVR exposure has pleotropic effects in the micro-environment of the skin, which can modulate various miRNAs and their target genes. Synergistically, the miRNAs can modulate the expression patterns of various genes in biologically relevant pathways linked to UVR-induced skin cancer.

## MATERIALS AND METHODS

### Mice and treatments

The mice used in the present study (WT and TNFα knock out) were on FVB background. The mice after genotyping were housed in groups of two to three in transparent plastic cages in light, humidity, and temperature-controlled rooms. The food and water were available *ad libitum*. The ages of the mice were 5-6 weeks at the time of experimentation. The dorsal hairs of the mice were shaved 3-4 days before experimentation. UVR source was Kodacel-filtered FS-40 sun lamps which comprises approximately 60% UVB and 40% UVA. Mice were exposed to UVR (2.0 kJ/m^2^) using a bank of six Kodacel-filtered sunlamps available in procedure rooms. UVR doses were routinely measured for its accuracy using a UVX radiometer. UVR lamps were purchased from National Biologicals Corporation, (Beachwood, OH), Kodacel filters from Unique Photo Inc. (Fairfield, NJ), and UVX-radiometer from UVP (Upland, CA). The details about the generation of TNFα KO mice are described elsewhere [16]. Similarly, the UVR-induced skin carcinogenesis protocol is explained earlier in detail [24]. All mice were depilated one day prior to UVR exposure. Mice from both groups were sacrificed 4 hrs post-UVR treatment. The animal protocols in the study were approved by the University of Wisconsin-Madison Research Animal Resources Committee in accordance with the National Institute of Health Guideline for the Care and Use of Laboratory Animals.

### RNA isolation, microRNA array profiling

Total RNA was isolated from the dorsal epidermal skin of the mice using the Trizol (Invitrogen, USA) method. The concentration of RNA was determined by measuring the absorbance at 260nm in a NanoDrop spectrophotometer. The quality of the total isolated RNA for microRNA profiling was evaluated by Exiqon Services, using an Agilent 2100 Bio analyzer (Agilent Technologies, Palo Alto, CA, USA). The integrity of the isolated RNA samples was confirmed on agarose gel. RNA samples from two mice (n=2 each) in each group were pooled for profiling.

MiRNA array profiling experiment was conducted at Exiqon Services (Denmark). Briefly, a total of 750 ng RNA from both sample and reference was labeled with Hy3™ and Hy5™ fluorescent label respectively, using the miRCURY LNA™ microRNA Hi-Power Labeling Kit, Hy3™/Hy5™. There were 1157 spots in triplicate on the miRNA array slide. The Hy3™ and Hy5™-labeled RNA samples were mixed pair-wise and hybridized to the miRCURY LNA™ microRNA Array 7th Gen, which contains capture probes targeting all registered mouse miRNAs in miRBASE 18.0. The hybridization was performed using a Tecan HS4800™ hybridization station (Tecan, Austria) and slides were scanned and stored in an ozone free environment in order to prevent bleaching of fluorescent dyes. The labelled slides were scanned in Agilent G2565BA Microarray Scanner (Agilent Technologies, Inc., USA) and analyzed using the ImaGene® 9. The quantified signals were background corrected (Normexp with offset value 10), [37] and normalized using the regression algorithm global Lowess (LOcally WEighted Scatterplot Smoothing). Principle Component Analysis (PCA) was performed for quality control.

### Primers for miRNA

To validate the miRNA profiling results, we have selected up- and down-regulated microRNAs and compared to their respective controls. The miRNAs chosen for validation having average Hy3 values (intensity of spot) >7, and log fold change values >1. All miRNA primers of mouse namely miR-31-5p, miR-196a-5p, miR-127-3p, miR-206-3p, miR-411-5p, miR-709, miR-322-5p, miR-32-5p, miR-33-5p, miR-376a-3p, miR-144-3p, and miR-136-5p were procured from Exiqon. U6 snRNA (mmu) was used as an internal control primer set. A detailed list of microRNA primers along with their target sequences is available online at Exiqon web site (http://www.exiqon.com/plate-layout-files).

### cDNA synthesis, real-time reverse transcriptase polymerase chain reaction (RT-PCR)

Reverse transcription reaction was set up using cDNA synthesis kit (Exiqon, miRCURY LNA™ Universal RT miRNA PCR, Polyadenylation and cDNA synthesis kit II product code 203301). Briefly, the template RNA (5ng/μL), 5X reaction buffer (2μL), enzyme mix (1μL), and nuclease free water was added together to make final volume to 10μL. For cDNA synthesis reaction, PCR settings were as follow: 60 min at 42°C, heat inactivation of reverse transcriptase for 5 min at 95°C and finally stored at 4°C.

Before using cDNA for RT-PCR reaction, the required amount of cDNA was diluted 80X (1:80 dilution) in nuclease free water and used immediately. For running the RT-PCR reaction, a working solution was prepared with ExiLENT SYBR Green (Exiqon, product code 203402) master mix (5 μL), PCR primer mix (1 μL), diluted cDNA template (4 μL) and the total volume was adjusted to 10uL. The real time PCR reaction was set up in Bio-Rad machine. The RT-PCR program was set up as follows: 95°C for 10 min, 40 amplification cycles at 95°C, 10sec at 60°C, 1 min ramp rate 1.6C/sec.

### Analysis of microRNA expression in real time

To determine the fold change analysis in our mouse samples, we have calculated the differences in Ct values of our endogenous control (U6 snRNA) and miRNA samples. Briefly, first the Ct values for all the samples are extracted and delta Ct is calculated as the difference in Ct between microRNA target and endogenous control [ΔC_T_ = C_T_ (target miRNA) - C_T_ (endogenous control)]. Secondly, the ΔΔC_T_ is calculated [ΔΔC_T_ = ΔC_T_ (sample of interest) - ΔC_T_ (control sample)]. Normalization of target miRNA target gene expression in the sample of interest is determined as 2^−ΔΔCT^. At final, the normalized expression level of control sample is set to 1 and change in target microRNA gene expression is determined as: Fold change in target microRNA expression = 1- normalized target miRNA expression in sample of interest.

### Bioinformatics tools for target prediction and pathway analysis

For prediction of miRNA targets, we used freely available bioinformatics tools such as DNA intelligent analysis or DIANA (http://diana.cslab.ece.ntua.gr/), MIRANDA, Target Scan (http://www.targetscan.org/), and miRNA database or miRDB (http://www.mirbase.org/). These bioinformatics tools predict the miRNA and its putative targets on the basis of sequence complementarity in 5′ and 3′ UTRs along with various sequence based mathematical algorithms. We also used DIANA miRPath v.2.0 for investigating the combinatorial effects of UVR-induced microRNAs in various biologically relevant pathways [38]. For pathway analysis a threshold p-value 0.05 along with MicroT threshold 0.8 were used for both up- and down-regulated microRNAs. We have selected pathways union mode, which identifies all the significantly targeted pathways by the selected miRNAs. In brief, we used the DIANA-miRPath web-server to predict miRNA targets (in coding or 3′-UTR regions) using the DIANA-microT-CDS algorithm. We used the default settings (p value 0.05, and threshold score 0.8), which returned an average of 350 targets per miRNA. These observed predicted and/or validated interactions were subsequently combined with sophisticated merging and meta-analysis algorithms [38]. Information about the chromosomal localization of miRNA was obtained from miRDB.

### Statistical analysis

Limma [39] and differential expression analysis [40] packages were used to determine differentially expressed genes in the following comparisons: WT vs. WT+UVR, WT vs. TNFα KO, TNFα KO vs. TNFα KO+UVR, and TNFα KO+UVR vs. WT+UVR. Adjusted p-value < 0.05 and |log_2_ fold-change (FC)| > 1 were used as cut-off criteria. Differences in expression level of miRNAs in uninvolved skin (skin without tumor) and SCC were examined using unpaired t-test. p≤ 0.05 was considered to be statistically significant.

## SUPPLEMENTARY MATERIAL TABLES



## Abbreviations

TNFα: tumor necrosis factor alpha
UVR: ultraviolet radiation
SCC: squamous cell carcinoma
