# Supplementary material for: Ultraviolet radiation-induced tumor necrosis factor alpha, which is linked to the development of cutaneous SCC, modulates differential epidermal microRNAs expression
Source: Oncotarget. 2016 Feb 22;7(14):17945–56. doi: 10.18632/oncotarget.7595 (PMC4951262; doi:10.18632/oncotarget.7595)
Supplement: Supplementary file 1 [file oncotarget-07-17945-s001.pdf]

Ultraviolet radiation-induced tumor necrosis factor alpha, which is linked to the development of cutaneous SCC, modulates differential epidermal microRNAs expression

Supplementary Material

S1 Table: List of genes found to be associated with various miRNA in DIANA (DNA intelligent analysis), MIRANDA, and Target Scan online databases.

miRNA name (mmu)	Predicted in DIANA	Predicted in MIRANDA	Predicted in TARGET SCAN
**miR-196a-5p**	Hoxc8, Epc2, Pbx1, Zbtb26, Prtg, Lin28b, Prlr, Slc9a6, Gan, Tmem194, Lcor, Igf2bp1, P4ha2, Hoxa7	Hoxc8, Epc2, Pbx1, Zbtb26, Prtg, Lin28b, Prlr, Slc9a6, Gan, Tmem194, Lcor	Hoxc8, Epc2, Pbx1, Zbtb26, Prtg, Lin28b, Prlr, Slc9a6, Gan, Tmem194, Igf2bp1
**miR-196b-5p**	Hoxa7, Epc2, Hoxc8, Igf2bp1, Nr6a1, Zbtb26, Prtg, Lin28b, Hmga2, Prlr, Slc9a6, Gan, Pbx1, Tmem194	Hoxa7, Epc2, Hoxc8, Igf2bp1, Nr6a1, Zbtb26, Prtg, Lin28b, Hmga2, Prlr, Slc9a6, Gan, Pbx1, Tmem194	Epc2, Hoxc8, Igf2bp1, Nr6a1, Zbtb26, Prtg, Lin28b, Hmga2, Prlr, Slc9a6, Gan, Pbx1, Tmem194
**miR-377-3p**	Rnf38, Nudt11, Anapc4, Setbp1, Zfp516, Celf2, Ube2d2, Zfp148, Phf6	Rnf38, Nudt11, Celf2, Ube2d2, Zfp148, Phf6	Rnf38, Nudt11, Setbp1, Celf2, Ube2d2, Zfp148, Phf6
**miR-691**	Bcl11b, Etv3, Tnrc6b, Zfp451, Celf2, Cntn1, **Mmp16**, Zfp618, Sp9, Usp32, Sp1, Pitpnc1, Ccdc117	Bcl11b, Etv3, Tnrc6b, Zfp451, Celf2, Cntn1, Mmp16, Zfp618, Usp32, Sp1, Pitpnc1, Ccdc117	None
**miR-206-3p**	Foxp1, **Gja1**, Crebl2, Coro1c, Slc44a1, Lrch1, AW549877, Hnrnpa3, Zfyve27, **Bdnf**, Hivep3, Fndc3a, Rnf165, **Ptplad1**, Ust, Serp1, G6pdx, Twf1, Zfp36l2, **Mmd**, **Pax7**, Fndc3b, Tmsb4x, Pdik1l, Ptprg	Foxp1, **Gja1**, Crebl2, Coro1c, Slc44a1, Lrch1, AW549877, Hnrnpa3, Zfyve27, **Bdnf**, Hivep3, Fndc3a, Rnf165, **Ptplad1**, Ust, Serp1, G6pdx, Twf1, Zfp36l2, Mmd, **Pax7**, Fndc3b, Tmsb4x, Pdik1l, Ptprg	Foxp1, **Gja1**, Crebl2, Coro1c, Slc44a1, Lrch1, AW549877, Hnrnpa3, Zfyve27, **Bdnf,** Hivep3, Fndc3a, Rnf165, **Ptplad1**, Ust, Serp1, G6pdx, Twf1, Zfp36l2, **Mmd**, **Pax7**, Fndc3b, Tmsb4x, Pdik1l, Ptprg **(Green bold = experimentally validated**

**miR-3065-3p**	Lox, Nfatc3, Dot1l, AI593442, Adamts17, Slc30a3, Nav3, Ing4, Gulp1, Col7a1, Nasp, Unc45a, Fubp1	None	None
**miR-322-5p**	Ubfd1, Atxn7l3b, Luzp1, Atxn7l3, Spryd3, Rasgef1b, Prdm11, Sox6, Pappa, Rfx3, Akt3, Rnf111, Shoc2, Mybl1, Rbms1, Apln, Col12a1, Clock, Bcl2l2, Lhx4, Zdhhc24, Eif2c1, Pcmt1, Ski, Wdtc1, Dll1, Eif2c4, Ptprr, Cc2d1b, Myt1l	Ubfd1, Luzp1, Atxn7l3, Spryd3, Rasgef1b, Sox6, Pappa, Rfx3, Akt3, Rnf111, Shoc2, Apln, **Col12a1**, Clock, Bcl2l2, Lhx4, Zdhhc24, Pcmt1, Ski, Wdtc1, Dll1, Eif2c4, Ptprr, Cc2d1b, Myt1l	Ubfd1, Atxn7l3b, Luzp1, Atxn7l3, Spryd3, Rasgef1b, Prdm11, Sox6, Pappa, Rfx3, Akt3, Rnf111, Shoc2, Mybl1, Rbms1, Apln, Col12a1, Clock, Bcl2l2, Lhx4, Zdhhc24, Eif2c1, Pcmt1, Ski, Wdtc1, Dll1, Eif2c4, Ptprr, Cc2d1b, Myt1l
**miR-31-5p**	Satb2, Ppp3ca	Satb2, Ppp3ca	Satb2, Ppp3ca
**miR-335-5p**	Ahsa2, Dync2h1, Nckap5	Ahsa2, Dync2h1, Nckap5	Ahsa2, Dync2h1, Nckap5
**miR-335-5p**	Ahsa2, Dync2h1, Nckap5	Ahsa2, Dync2h1, Nckap5	Ahsa2, Dync2h1, Nckap5
**miR-379-5p**	Lphn3, Sp3tc1	Lphn3	None
**miR-434-3p**	Mybpc3, Lin7a, Sp9, Ccnl1	Mybpc3	None
**miR-511-3p**	Sema3a, Bbx, Galnt7	None	None
**miR-5617-5p**	Rfx3, Pbx3	None	None
**miR-379-5p**	Lphn3, Sh3tc1	Lphn3	None
**miR-31-3p**	Nfia	None	None
**miR-136-5p**	Cpeb2	None	None
**miR-411-5p**	Maml3	None	None
**miR-709**	Mbd6	None	None
**miR-185-3p**	R3hdm2	None	None
**miR-136-3p, miR-127-3p, miR-376a-3p, miR-434-5p, miR-376b-5p, miR-434-5p, miR-541-5p**	No interaction found	---	---

S2 Table: miRNA target identified on miRDB.

**Target Rank (left to right)**	**Target Score**	**miRNA Name (mmu)**	**Target Gene Symbol (target score high to low) on miRDB**
1 to 11	80-99	miR-196a-5p	Hoxc8, Zmynd11, Ccdc47, Rcc2, Rgl2, Slc9a6, Hoxb7, Neto2, St6galnac3, Epc2, Trp53bp2,
1 to 11	>80-99	miR-196b-5p	Hoxc8, Zmynd11, Ccdc47, Rcc2, Rgl2, , Neto2, Epc2, Trp53bp2, St6galnac3, Hoxb7,Slc9a6
**1 to 22**	**>80-99**	**miR-31-5p**	**PKCE,** Rsbn1, Slc1a2, Mbnl3, Rhbdl3, Ovca2, Jazf1, Scn2a1, 4921501E09Rik, **Ppp1r3d**, Pik3c2a, Cpsf2, Depdc5, Ispd, Zfp799, Slc6a6, Elovl7, Igsf11, Ikzf1, Pcgf6, Cntln, Gpd1l, Serpinb5
1 to 8	>80-93	miR-31-3p	Kif1b, Klhl31, Lrrc42, Tbc1d30, Pvr, Zfp2, Yes1, Frs2
1 to 13	>80-91	miR-136-5p	Mtmr4, Chrdl1, Braf, Trpc4ap, Gria1, Mdm1, Taf7, Etf1, Zfp532, Rpusd4, Mab21l1, Atrn, Mid2
1 to 8	>80-91	miR-411-5p	Elfn1, Eif4g2, St18, Cwf19l2, Irf2bp2, Ptx3, Nfatc2ip, Smarca2
1	86	miR-434-5p	Cacna2d2
1 to 6	>80-100	miR-379-5p	Rfng (**target score=100**), Fbxl20 (**target score=100**),Themis , Eif4g2, Tex2, Ube2e3
1 to 48	>80-100	miR-709	Armc9 (**target score=100**), Cbx5, Zfyve27, Coro2b, Leng8, Cbfa2t3, Lnp, Mid1, Nrf1, Ptpn7, A630055G03Rik, D330012F22Rik, Iqce, Rnf213, Cntn2, Nav1, Adora2b, Ypel2, Onecut3, Tubb5, Myst2, Psmb11, Rg9mtd2, Dixdc1, Cbfa2t2, **Myc**, A430033K04Rik, Fem1a, Serpina3n, Stk39, 9830001H06Rik, Rnf165, Gga2, Scube1, 1110028C15Rik, Fbxl17, Trp53i11, Ap3m1, Stox2, Ncoa3, Tpbg, Tmem179, Slc9a8, Mbd4, Dlgap1, Plxna2, Naa50, Usp22
0	>80	miR-136-3p	None
0	>80	miR-127-3p	None
0	>80	miR-376a-3p	None
Target Rank (left to right)	Target Score	miRNA Name	Target Gene Symbol (target score high to low) on miRDB
1 to 19	>80-90	miR-335-5p	BC003965, Enpp4, Nt5dc3, Mtap6, Rab3c, Hnrnpr, Wdr26, Homez, Thap2, Rab11b, Gm608, Sorcs1, Etf1, Pole4, B4galt4, Efr3a, Nrxn1, Commd2, C430048L16Rik

1 to 10	>80-88	miR-434-3p	Tmem207, Cyp26b1, Nck1, Gem, Pla2g16, Dcaf6, Snurf, Snrpn, 4933403F05Rik, Rnf139
1 to 60	>80-99	miR-377-3p	Rnf38, Thsd7a, Trip12, Zbtb4, St8sia4, Tmx1, Grsf1, Zadh2, Pitx2, Mga, Bend6, Aqp4, Nudcd2, Cdon, Cul1, Zfp187, Bhlhe23, Larp4, B230219D22Rik, Rasa1, Armc8, Phf6, Paxip1, Fbxl17, Ncoa6, Kcnj8, Slc6a19, Npsr1, Stk35, Agpat4, Arid4b, Btg3, Hspbap1, Sumf1, Ube2h, Nip7, 6720489N17Rik, Rsbn1, Slc7a11, Dnajb9, Xiap, Shisa5, Bend3, Rfx3, Cpsf6, Nt5c3l, Nts, Bcl2l1, Zfp462, Pafah1b2, Areg, Plcb1, Hexim1, A730037C10Rik, Gas2l3, Nudt11, Crebl2, Ghitm, Wdr6, Zfp148
1 to 61	>80-99	miR-511-3p	Treml2, Sema3a, Tjp1, Tnrc6b, Tmem229a, Camk4, Nbeal1, Lpp, Tank, Colec10, Mapk6, Pank4, Golt1a, Fech, Slc28a3, Dcun1d3, Bbx, Rock2, Aktip, Fuca2, Srp54a, Zfp192, Pofut1, Gabrb3, Plscr1, Nox4, Cdc14a, Pou2f1, Sap30, Zfp641, 9430020K01Rik, Fndc3b, 41524, Xpo7, Tfap2b, Baz1a, Dcaf17, Tnfrsf21, Gm8369, Ncam2, Ccdc125, Fbxo5, Slc5a7, Rnasel, Dclre1b, Eda2r, Ddx17, LOC100045026, Pum1, Adamts5, D630023F18Rik, Slc7a11, Osbpl6, Ugt1a9, Txlnb, Ugt1a1, Ugt1a2, Ugt1a6a, Ugt1a5, Ugt1a10, Ugt1a7c
1 to 19	>80-96	miR-5617-5p	8030462N17Rik, Rsf1, D4Ertd22e, Pkn2, Ccr4, Ube2j2, Gria2, BC068281, Neurl4, Pafah1b1, Fgf13, Fam175b, Mapk1ip1l, Edem2, Slc6a14, Fut9, Tnrc6b, Hecw2, Arid2
1 to 59	>80-95	miR-691	Plod2, Rod1, Nhsl2, B230209E15Rik, A730037C10Rik, Homer2, Csrp2bp, Cdr1, Actl6a, Foxi2, Slc4a8, Bnc1, Tmem55a, Tmem229a, Gbas, Gtf2h1, Tmem184b, Kcnq5, Dram2, D3Ertd751e, Sult1b1, Snurf, Snd1, Hdlbp, Cnpy1, N4bp2l2, Fam175b, Tctn3, Ube2g1, Slc16a5, Lpp, Syn3, Cndp1, C330027C09Rik, Top1, Tmcc3, Pcdh17, Gm11992, Ccdc117, Trh, Ccnt2, Rbm18, D19Ertd737e, Erp44, Gm7969, Tob2, LOC100505143, Mfsd1, Col19a1, Ubn2, Bcl11b, Adamts16, Gramd3, Camsap2, Epha5, Slc17a8, Tmem167, Klhl24, Cpsf2
1 to 19	>80-96	miR-154-5p	Kctd14, Cul2, Ubtd2, Mmp20, Dock1, Sos2, Msl2, Sp4, Chm, Serpinb2, Cog7, Ppp2r2a, Cpne4, Rsph4a, E2f5, Myst2, Asap2, Eps15, Qk
1 to 46	>80-97	miR-206-3p	Rufy2, Sri, Rfesd, Col4a3, Kalrn, Gja1, Entpd7, Ankib1, Bdnf, Coro1c, Ets1, Ankrd29, Gnpda2, Lrrfip1, Kif2a, Tbc1d15, Edn1, Zfp280d, Cmpk2, Rit2, Arcn1, Serp1, Rsbn1, Snx2, C77370, 1110003E01Rik, Wdr48, Fam150b, Hmcn1, Slc29a3, Pax3, Rrbp1, Meox2, Fbxo33, 2410075B13Rik, Ptpn14, Cebpz, Foxp1, C1galt1, Smarcb1, Hnrnpa3, Dcaf12l1, Igf1, Fndc3a, Slc35a5, Pde7a
1 to 22	>80-92	miR-376b-5p	Tigd4, Zfp1, Eif2ak2, Bnip3l, Cacnb4, Heca, Sfxn1, Fbxl3, Ptgs2,Ttc9c, Acss1, Syne1, 1110067D22Rik, Gm609, Hsf3, Bclaf1, Magi2, 4930417G10Rik, Prrx1, Rgs5, AI593442, Hrasls

1 to 20	>80-97	miR-185-3p	Nr5a1, Cbfa2t3, Vamp2, Vat1, Tnrc6b, Gm10862, Ehd1, Fam57b, Cadm3, Kcnk3, Ptpn1, Add1, Fmod, Mboat4, Unc13a, Wscd2, Zfp236, Tusc5, Vipr1, Rnf165
1 to 6	100	miR-379-5p	Rfng (target score=100), Fbxl20 (target score=100), Themis, Eif4g2, Tex2, Ube2e3
1 to 8	>80-91	miR-411-5p	Elfn1, Eif4g2, St18, Cwf19l2, Irf2bp2, Ptx3, Nfatc2ip, Smarca2
1	86	miR-434-5p	Cacna2d2
1 to 24	>80-98	miR-3065-3p	Fam135a, Xkr6, Ubtd2, Adamts15, Aplf, Wac, Smek2, Camk2g, Lsm14a, Dio2, 3830406C13Rik, Nfia, Palm2, Il1rapl2, Ppm1d, Ankrd49, Scn9a, Hmgcs1, Dusp22, Ric8, Blmh, Camsap1, Stag2, Mest
1 to 11	>80-87	miR-541-5p	Grm7, Lysmd3, Kcnmb2, Acvr1b, Gab1, A830010M20Rik, Stk3, Hoxd10, Paqr5, Zfx, Plaa
1 to 113	>80-100	miR-322-5p	Mapkap1 (target score=100), Fgd4, Btrc, N4bp1, Strbp, Pacsin2, Tacc1, Ankib1, Lrp6, Hsd17b7, Etnk1, Gm10220, Vapb, 5031410I06Rik, Kl, Rasgef1b, Aff4, Usp14, Tbl1xr1, Luzp1, Mapk8, 1700025G04Rik, Fbxl20, Wee1, Ubn2, Tlk1, 2700078E11Rik, Myt1l, Crebl2, Srpr, Kif5c, Cds2, Phf19, Wipi2, Ttc14, Ahcyl2, Pldn, Ccnt2, Rnf10, Kif21a, Actr2, Plekhh1, Eif3a, Cask, Sall1, Onecut2, Prkar2a, Col5a1, Csde1, Zik1, 2310008H09Rik, Pappa, Hus1, Plcxd2, Zfp592, Nfatc3, Ubfd1, Cd2ap, Mgat4a, Mtmr3, Dclk1, Ssr1, Nuak2, Fam164a, Lrp2, Ash1l, Reep1, Capza2, Zfp609, Mob4, Abhd13, Phc3, Gm10471, Mkks, Exoc8, Ccne1, Kif23, Lats1, Nup210, Tbx22, Cpd, Tmem135, Traf3, Ppp6c, Slc20a2, Ddx3x, Mfn2, Slc4a8, Cdc25a, Lats2, Zbtb5, Polr3f, Stxbp3a, Cdc14b, Col4a3bp, Rab9b, Atp7a, Sgk1, Col24a1, Eya1, Ppm1d, Gpatch8, Nf1, Fam133b, Rad23b, Entpd7, Ywhah, LOC100504975, Avl9, Ppap2a, Slc1a4, 2400003C14Rik, Fshb
